# Genetic polymorphisms and phylogenetic analyses of the Ü-Tsang Tibetan from Lhasa based on 30 slowly and moderately mutated Y-STR loci

**DOI:** 10.1080/20961790.2020.1810882

**Published:** 2020-10-12

**Authors:** Jiuyang Ding, Haoliang Fan, Yongsong Zhou, Zhuo Wang, Xiao Wang, Xuheng Song, Bofeng Zhu, Pingming Qiu

**Affiliations:** a Shanghai Key Laboratory of Forensic Medicine, Shanghai Forensic Service Platform, Academy of Forensic Science, Shanghai, China; b School of Forensic Medicine, Southern Medical University, Guangzhou, China; c School of Basic Medicine and Life Science, Hainan Medical University, Haikou, China; d Multi-Omics Innovative Research Center of Forensic Identification, Department of Forensic Genetics, School of Forensic Medicine, Southern Medical University, Guangzhou, China; e Department of Psychiatry, The First Clinical Medical College, Shanxi Medical University, Taiyuan, China; f Key Laboratory of Shaanxi Province for Craniofacial Precision Medicine Research, College of Stomatology, Xi’an Jiaotong University, Xi’an, China; g Clinical Research Center of Shaanxi Province for Dental and Maxillofacial Diseases, College of Stomatology, Xi’an Jiaotong University, Xi’an, China

**Keywords:** Forensic sciences, forensic genetics, Y-STR, Ü-Tsang Tibetan, phylogenetic analysis, AGCU Y30, O-M122

## Abstract

As a result of the expansion of old Tibet on the Qinghai-Tibet Plateau, Tibetans diverged into three main branches, Ü-Tsang, Amdo, and Kham Tibetan. Ü-Tsang Tibetans are geographically distributed across the wide central and western portions of the Qinghai-Tibet Plateau while Lhasa is the central gathering place for Tibetan culture. The AGCU Y30, a 6-dye fluorescence kit including 30 slowly and moderately mutated Y-STR loci, has been validated for its stability and sensitivity in different biomaterials and diverse Chinese populations (Han and other minorities), and widely used in the practical work of forensic science. However, the 30 Y-STR profiling of Tibetan, especially for Ü-Tsang Tibetan, were insufficient. We utilized the AGCU Y30 to genotype 577 Ü-Tsang Tibetan unrelated males from Lhasa in the Tibet Autonomous Region of China to fill up the full and accurate Y-STR profiles. A total of 552 haplotypes were observed, 536 (97.10%) of which were unique. One hundred and ninety-four alleles were observed at 26 single copy loci and the allelic frequencies ranged from 0.0017 to 0.8180. For the two multi-copy loci DYS385a/b and DYS527a/b, 64 and 36 allelic combinations were observed, respectively. The gene diversity (GD) values ranged from 0.3079 at DYS391 to 0.9142 at DYS385a/b and the overall haplotype diversity (HD) was 0.9998, and its discrimination capacity (DC) was 0.9567. The population genetic analyses demonstrated that Lhasa Ü-Tsang Tibetan had close relationships with other Tibetan populations from Tibet and Qinghai, especially with Ü-Tsang Tibetan. From the perspective of Y haplogroups, the admixture of the southward Qiang people with dominant haplogroup O-M122 and the northward migrations of the initial settlers of East Asia with haplogroup D-M175 hinted the Sino-Tibetan homologous, thus, we could not ignore the gene flows with other Sino-Tibetan populations, especially for Han Chinese, to characterize the forensic genetic landscape of Tibetan.

## Introduction

The Qinghai-Tibet Plateau (the Himalayan Plateau), the largest plateau in China and the highest in the world, starts from the southern margin of the Himalayas to the northern margin of the Kunlong, Altun and Qilian Mountains, reaches the Pamir Plateau and the Har Goolun Range (Karakoram Range) in the west, and connects with the western part of the Qinling Mountains and the Loess Plateau in the east and northeast. The Qinghai-Tibet Plateau which is called the Roof of the World or the Third Pole is generally between 3 000 and 5 000 m above sea level with an average elevation exceeding 4 500 m. It is one of the origins of the Chinese nation and Chinese civilization, and the ancients in Chinese history, Fu Hsi, Yan Emperor, and Yu, were the ancient Qiang people in the plateau. With the environmental changes, population expansions and the developments of the agricultural society, the plateau residents were predominantly Tibetan population since the rise of the Tubo ancestors in the river valley of Yalong which located at Shannan Prefecture (Lhoka), which gradually formed the plateau culture system dominated by Tibetan culture [1].

The initial stage occupation of lower elevations in Qinghai-Tibet Plateau could be traced back to 40–25 thousand years ago (kya) by highly mobile foragers. They followed a logistical mobility pattern and focused on the collection of high ranked resources. Then the full-scale, year-round occupation of the upper regions of the plateau was occupied by early Neolithic pastoralists [2,3]. From the genetic perspective, the initial peopling and early colonization of the Qinghai-Tibet Plateau could trace back to about 62–38 kya by the shared ancestry of Tibetan-enriched sequences. The whole-genome sequencing results provided compelling evidence of the co-existence of Paleolithic and Neolithic ancestries in the Tibetan gene pool which diverged from that of Han Chinese about 15–9 kya [4]. The paternal Y-chromosomal and maternal mitochondrial lineages also demonstrated that anatomically modern humans in Tibet colonized the Qinghai-Tibet Plateau at Upper Paleolithic Age colonization and expanded rapidly at the Neolithic Age [5,6]. In the following primitive communes time for survival and expansion, Tibetans diverged three dominant embranchments, Ü-Tsang, Amdo, and Kham Tibetan, to find the appropriate environment to live long and prosper. In addition, they separately established their unique cultures with the original memory of the old Tibetans [7].

Ü-Tsang Tibetan, the previous Dbus-Gtsang before Ming Dynasty (AD 1368–1644), geographically covered the central and western portions of the Tibetan cultural area, including the Tsang-po (Gtsang-po) watershed, the western districts surrounding and extending past Mount Kailash, and much of the vast Chang Tang (Byang-thang) Plateau to the north. Lhasa (lā sà), the Sunshine City and the capital of Tibet Autonomous Region, was the economic, political, cultural, scientific and educational centers of Ü-Tsang since the establishment of the old Tubo Kingdom (AD 618–842) after Songtsen Gampo unified the Qinghai-Tibetan Plateau. The language of Ü-Tsang Tibetan is a branch of Tibetan language, which belongs to the Sino-Tibetan language family [8,9]. However, due to the geographic isolation and cultural differences, the three Tibetan branches, with dialects that all belong to Tibeto-Burman language, could not communicate with each other.

In the past decades, as a robust molecular tool, Y chromosomal short tandem repeat (Y-STR) markers are applied to investigate the genetic diversity, migration, and evolution of human populations with increasingly expanded potential applications [10–13]. Recently, a novel panel based on 30 slowly and moderately mutated Y-STR loci for forensic applications was developed and validated. The AGCU Y30 contains 30 loci labeled by six fluorescent dyes, including 17 Yfiler loci and 13 additional Y-STR loci (DYS549, DYS447, DYS388, DYS444, DYS460, DYS481, DYS520, DYS522, DYS643, DYS533, DYS557, and DYS527a/b) [14]. In addition, Tibetan populations of Lhasa, the key area for the diversification of Ü-Tsang Tibetan, have seldom been studied genetically. Therefore, we investigated the allelic and haplotype frequencies of 30 Y-STR loci in Ü-Tsang Tibetan population by 577 unrelated male individuals from Lhasa to fill the forensic genetic gaps. The purpose of this investigation is to assess the population and forensic applications of the 30 slowly and moderately mutated Y-STR loci and to evaluate the genetic relationships between Lhasa Ü-Tsang Tibetan (Lhasa Tibetan or Tibetan Lhasa, hereinafter) and other populations.

## Materials and methods

### Sample collection, PCR amplification, and genotyping

A batch of 577 buccal samples on FTA™ cards (GE Healthcare/Whatman, Buckinghamshire, UK) were collected from Lhasa unrelated male individuals in the Tibet Autonomous Region of China. The study was approved by the Ethics Committee of Xi’an Jiaotong University (No. XJTULAC201). All of the participants lived locally for three generations at least and signed the informed consent before sampling. The buccal samples were amplified using AGCU Y30 assay (AGCU Incorporation, Wuxi, China). The processes of PCR amplification and electrophoresis were performed according to the method described by our previous studies [14–16].

### Reference populations and previously reported Y-STR data

A total of 3 481 haplotypes from 21 previously reported populations [17–28] were selected for comparison through the 17 Y-STR loci (DYS456, DYS389I, DYS390, DYS389II, DYS458, DYS19, DYS385a/b, DYS393, DYS391, DYS439, DYS635, DYS392, YGATAH4, DYS437, DYS438, and DYS448). The genotype data of the 34 reference populations were obtained by the Yfiler®, Yfiler® Plus (Thermo Fisher Scientific, Waltham, MA, USA), PowerPlex® Y23 (Promega Corporation, Madison, WI, USA), AGCU Y24 (AGCU Incorporation) and other available commercial Y-STR kits.

### Quality control

The Y-STR typing experiments were performed strictly according to the recommendations of DNA Commission of the International Society of Forensic Genetics (ISFG) [29]. In the course of testing experiment, the UltraPure™ Distilled Water (Thermo Fisher Scientific) and the Control DNA 9948 (Promega Corporation) were used as negative and positive controls, respectively. The Center for Forensic Science of Southern Medical University has passed the laboratory accreditation of China National Accreditation Service for Conformity Assessment (CNAS). The data of 577 Lhasa Ü-Tsang Tibetan in present study were submitted to YHRD database and received the accession number of YA004692.

### Statistical and phylogenetic analyses

In this study, allele frequencies were calculated for every 30 Y-STR loci by direct counting. Arlequin Software v3.5 (http://cmpg.unibe.ch/software/arlequin3.5) was used to calculate haplotype frequencies. The forensic parameters, gene diversity (GD), haplotype diversity (HD), and discrimination capacity (DC) were calculated according to the formulas which were detailed described in our previous study [30,31]. The molecular variance analysis (AMOVA) were performed using the YHRD online tool (http://yhrd.org/pages/tools/amova) and the data were compared with publicly published populations. The Multi-Dimensional Scaling (MDS) was constructed on the basis of allele frequencies by means of SPSS 22.0 (IBM Corp., Armonk, NY, USA) based on population pairwise genetic distances (*R*_st_) between two populations with Euclidean distance [32]. The principal component analysis (PCA) was performed with R programming language based on the haplogroup frequencies, and used to explore the extent of correlation of genetic relationships. Additionally, phylogenetic relationships among different populations were conducted using the Molecular Evolutionary Genetics Analysis 7.0 (MEGA 7.0) software [33] by neighbour-joining (N-J) phylogenetic tree [34] based on *R*_st_ genetic distance matrix.

## Results and discussion

### Allele frequencies and forensic parameters

The allelic frequencies and GD values for the 30 Y-STR loci in Lhasa Ü-Tsang Tibetan were shown in Supplementary material Table S1. A total of 194 alleles were observed at 26 single copy loci and the allelic frequencies ranged from 0.0017 to 0.8180. For the two multi-copy loci DYS385a/b and DYS527a/b, 64 and 36 allelic combinations were observed, respectively. A high relatively level of genetic heterogeneity was exhibited in Lhasa Ü-Tsang Tibetan, with 28 out of 30 loci having the GD values greater than 0.5. The two loci with GD values less than 0.5 were DYS438 (0.4540) and DYS391 (0.3079), respectively. The multi-copy locus DYS385a/b displayed the highest GD (0.9142), followed by DYS527a/b (0.9103) and DYS481 (0.8308). In total, 14 microvariants were detected at DYS447, DYS448, DYS520, DYS385a/b, and DYS527a/b loci, and the microvariants were confirmed by re-amplification process.

As shown in Supplementary material Table S2, a total of 552 haplotypes were obtained at the 30 loci (AGCU Y30), of which 536 (97.10%) were unique, 10 (1.81%) were observed twice (H007-H016), four (H003-H006) occurred thrice, one (H002) and one (H001) were observed by four and five individuals, respectively. The overall HD was calculated to be 0.9998 with a DC of 0.9567 which demonstrated that the 30 Y-STR loci are valuable for forensic applications in Lhasa Ü-Tsang Tibetan.

### Genetic differentiation among populations

The pairwise *R*_st_ and corresponding *P* values between the Lhasa Tibetan and Anhui Han population, four Chinese Tibetan populations and other 16 minority ethnic groups based on 17 Y-STR loci were presented in Supplementary material Table S3. The results illustrated that significant differences were observed when comparing Lhasa Tibetan with other reference populations in China (*P* < 0.05). The Lhasa Ü-Tsang Tibetan had been found to be closely related to Ü-Tsang Tibetan (*R*_st_ = 0.0167) and Amdo Tibetan (*R*_st_ = 0.0260). Among 21 reference ethnic groups, the Lhasa Tibetan population was farthest to the Liannan Yao (*R*_st_ = 0.2297), followed by Gansu Kazakh (*R*_st_ = 0.2217). The largest genetic distance at 17 loci Y-STR haplotypes was observed between Liannan Yao and Gansu Kazakh (*R*_st_ = 0.6345), while the smallest was found between Yunnan Bai and Anhui Han (*R*_st_ = 0.0022).

**Figure 1.**
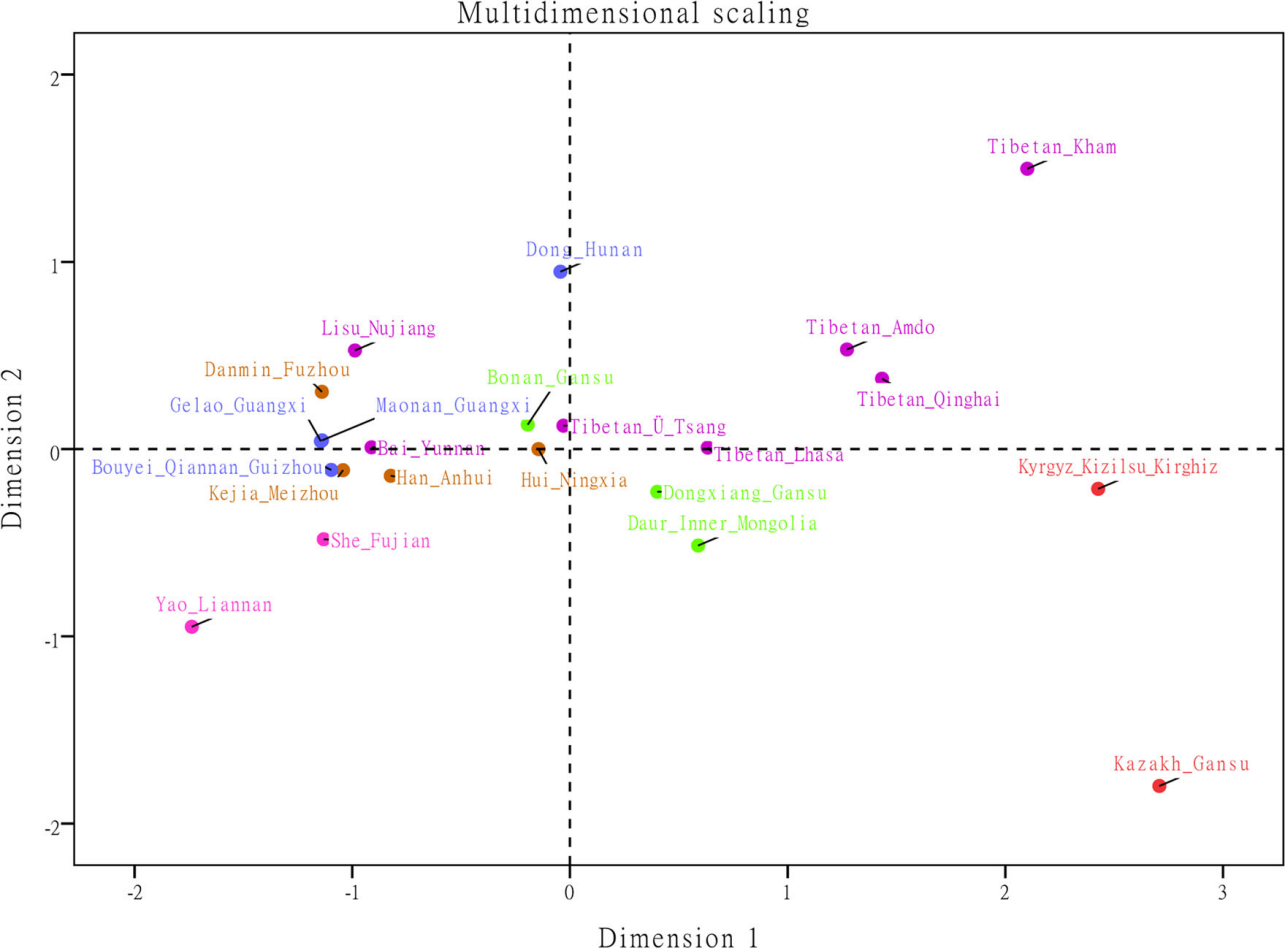
Multidimensional Scaling plots of our studied Lhasa Tibetan and 21 reference national populations based on Yfiler haplotypes.

**Figure 2.**
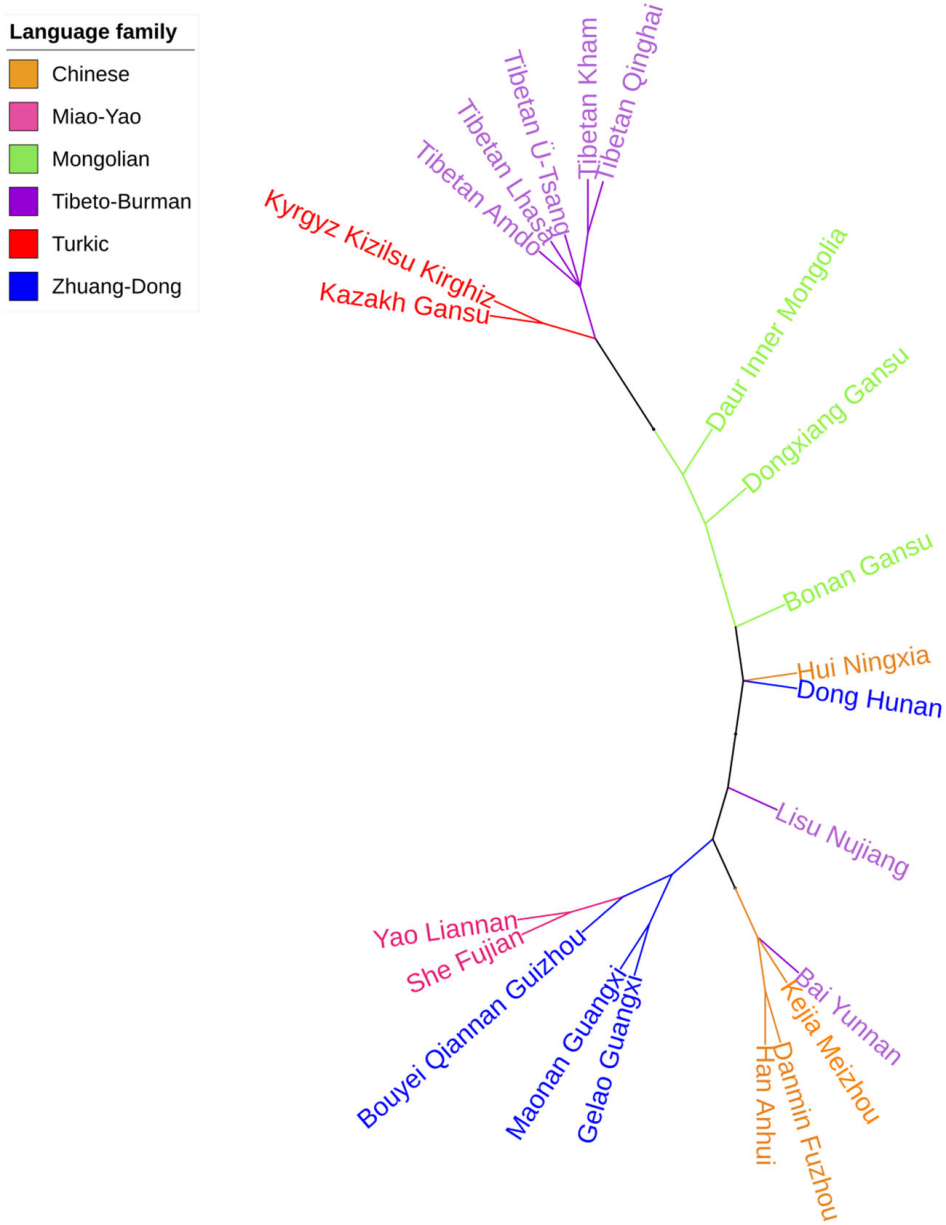
The N-J phylogenetic tree exhibited the genetic relationships between the Lhasa Tibetan and other 21 Chinese populations in different administrative divisions of China.

The phylogenetic relationships between the Lhasa Ü-Tsang Tibetan and 21 reference populations were displayed in MDS plot (Figure 1) and N-J tree (Figure 2). The Lhasa Ü-Tsang Tibetan clustered loosely in the middle of the MDS plot, together with four Chinese Tibetan populations from Ü-Tsang, Amdo, Kham and Qinghai. This result demonstrated the uniquely genetic features of the Tibetan population, which was consistent with the findings of previous autosomal chromosomal STR [35] and Y-STR studies [36]. Anhui Han closely clustered within the left center of the MDS, with several southern minority ethnic groups including Yunan Bai, Meizhou Kejia, Fuzhou Danmin, Guangxi Gelao, Guangxi Maonan, Guizhou Bouyei, Nujiang Lisu, and Fujian She, possibly due to the geographical location of these populations close to each other.

As illustrated in Figure 2, the Lhasa Tibetan and the four reference populations clustered in the same clade. The Lhasa, Ü-Tsang and Amdo shared one node of the branch, however, the Kham and Qinghai shared another node. The clustering characteristics were consistent with the cultures, religious belief and geographic locations of the Tibetan populations [28]. Although the Tibetan populations form a sister group with Kazakh and Kyrgyz in the N-J tree, the MDS plot displayed that the Kazakh possessed weaker genetic ties with Tibetan populations. Moreover, most of the minority ethnic groups from the south of China shared the same clade on the N-J tree, very similar to the distribution of these populations in the MDS plot. In general, the phylogenetic relationships constructed by the N-J dendrogram in accordance with the distribution pattern of the populations in the MDS plot.

### Y-haplogroup estimation

To clear up the detailed relationship between the Lhasa Ü-Tsang Tibetan population and other populations with diverse language families, the PCA was performed among 194 populations (in total 10 721 individuals) which included Austro-Asiatic, Austronesian, Altaic, Semito-Hamitic, Caucasian, Uralic, Dravidian, Indo-European, Tai-Kadai, Hmong-Mien, Tibeto-Burman, and Chinese (Southern and Northern Han) populations all over the world [37–50]. We used our in-house dataset, including 37 754 pieces of Y SNP/STR data and 109 142 Y-STR in total which mainly from Asia, to make predictions for Lhasa Ü-Tsang Tibetan in this study [51]. We selected the Y-STR data of 577 Lhasa Tibetan males in this study for the estimation of Y-haplogroups of Ü-Tsang Tibetan population. Eventually, 545 out of the 577 genotyped Y-STRs (94.45%) were observed in the derived state, thus defining nine haplogroups observed in Ü-Tsang Tibetan samples, belonging to major clades D and O2. The predominant haplogroups were O2-M122 (34.14%), D*-M174 (30.69%), D1-M175 (27.57%), C-M216 (2.42%), N-M231 (2.04%), J-M304 (1.79%), and Q-M242 (1.35%) (determined according to ISOGG 2019, https://isogg.org/tree/). Our results were in accordance with Rowold’s study [47], but G, H, R1, and R2 were not found in our study, while, the haplogroups of them totally accounted for 0.0654 in Rowold’s study of 153 Ü-Tsang samples [47].

**Figure 3.**
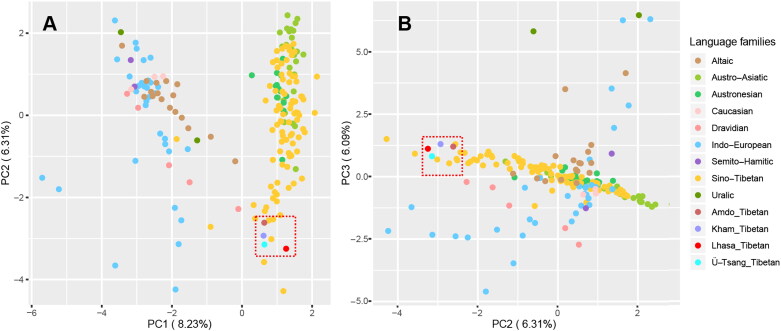
Principal component analysis (PCA) based on Y-haplogroup frequencies in 194 populations. (A) The PCA of PC1 and PC2. (B) The PCA of PC2 and PC3.

As shown in Figure 3, the first three components (20.63% totally) which accounted the proportions of the total variances observed within these populations were 8.23%, 6.31%, and 6.09%, respectively. The populations with different language families were clustered together, and Lhasa Ü-Tsang Tibetan had close relationships with Ü-Tsang Tibetan and other Tibetan populations (Kham and Amdo Tibetans), which clustered with Sino-Tibetan populations.

The Sino-Tibetan languages are a family of more than 400 languages, including two subfamilies, namely Tibeto-Burman and Chinese. The linguistic affinity between Tibeto-Burman and Chinese are well established with many cognates between Proto-Tibeto-Burman and Old Chinese [52]. The split time for Tibeto-Burman and Chinese was estimated around 6 kya based on lexical evidence and cladistic methods [53]. The O2-M122 had the highest frequency in East Asians, especially in Han Chinese (52.06% in northern Han and 53.72% in southern Han), and was also quite frequent in Hmong-Mien (51.41%) and Austronesian (26.31%) populations, but it is absent outside East Asia. From the perspective of Y haplogroups, Tibetan and Han Chinese are homologous, and Ü-Tsang Tibetan are an admixture of the southward Qiang people with dominant haplogroup O-M122 through a series of migrations since the Neolithic Age and the northward migrations of the initial settlers of East Asia with haplogroup D-M175 in the Late Paleolithic age [6,54–57].

## Conclusion

In summary, we investigated the genetic polymorphisms and forensic parameters based on 30 Y-STR loci in Ü-Tsang Tibetan from Lhasa, which demonstrated that the AGCU Y30 analyzed here were highly polymorphic in Lhasa Ü-Tsang Tibetan population and could be conducted in forensic applications. The phylogenetic analyses revealed the Lhasa Ü-Tsang Tibetan population had its unique genetic characteristics, and had close relationships with other Tibetan populations from Tibet and Qinghai. From the perspective of Y haplogroups, the admixture of the southward Qiang people with dominant haplogroup O-M122 and the northward migrations of the initial settlers of East Asia with haplogroup D-M175 hinted the Sino-Tibetan homologous, thus, we could not ignore the gene flows with other Sino-Tibetan populations, especially for Han Chinese, to characterize the forensic genetic landscape of Tibetan. In conclusion, the 30 Y-STR profiling of the Ü-Tsang Tibetan in Lhasa has potential applications for population genetics and forensic scenarios.

## Supplementary Material

Supplemental Material
